# Gastrodin Alleviates Tau Pathology by Targeting the Alzheimer's Risk Gene FERMT2, Reversing the Reduction in Brain Viscoelasticity

**DOI:** 10.1111/cns.70283

**Published:** 2025-03-22

**Authors:** Li Wang, Bo Li, Zhi Tang, Yang Wang, Yaqian Peng, Ting Sun, Anni Zhang, Xiaolan Qi

**Affiliations:** ^1^ Key Laboratory of Endemic and Ethnic Diseases, Ministry of Education and Key Laboratory of Medical Molecular Biology of Guizhou Province Key Laboratory of Molecular Biology of Guizhou Medical University Guiyang China; ^2^ School of Nursing Guizhou Medical University Guiyang China; ^3^ The Department of Imaging Affiliated Hospital of Guizhou Medical University Guiyang China; ^4^ The Department of Neurology Affiliated Hospital of Guizhou Medical University Guiyang China; ^5^ Collaborative Innovation Center for Prevention and Control of Endemic and Ethnic Regional Diseases Constructed by the Province and Ministry Guiyang China

**Keywords:** Alzheimer's disease, BBB, FERMT2, Gastrodin, matrix viscoelasticity, neuroinflammation, tau

## Abstract

**Background:**

The pathogenesis of Alzheimer's disease (AD) remains incompletely elucidated, and there is a notable deficiency in effective and safe therapeutic interventions. The influence of brain matrix viscoelasticity on the progression of AD has frequently been underestimated. It is imperative to elucidate these overlooked pathogenic factors and to innovate novel therapeutic strategies for AD. Gastrodin, a bioactive constituent derived from the traditional Chinese medicinal herb Gastrodia elata, exhibits a range of pharmacological properties, notably in the enhancement of neural function. Nevertheless, the underlying mechanisms of its action remain insufficiently elucidated. Consequently, this study seeks to examine the therapeutic effects and underlying mechanisms of gastrodin in the context of AD, with particular emphasis on its potential influence on the viscoelastic properties of the brain matrix.

**Methods:**

This study employs a range of methodologies, including the Morris water maze test, Y‐maze spontaneous alternation test, atomic force microscopy (AFM), immunofluorescence, transmission electron microscopy, molecular docking, and Cellular Thermal Shift Assay (CETSA), to demonstrate that gastrodin mitigates tau pathology by modulating FERMT2, thereby reversing the deterioration of mechanical viscoelasticity in the brain.

**Results:**

Gastrodin administration via gavage has been demonstrated to mitigate cognitive decline associated with AD, attenuate the hyperphosphorylation of tau protein in the hippocampus and cortex, and ameliorate synaptic damage. Additionally, gastrodin was observed to counteract the reduction in brain matrix viscoelasticity in 3xTg‐AD mice, as evidenced by the upregulation of extracellular matrix components pertinent to viscoelasticity, notably collagen types I and IV. Furthermore, molecular docking and CETSA revealed a strong binding affinity between gastrodin and FERMT2. Gastrodin treatment resulted in a reduction of FERMT2 fluorescence intensity, which is selectively expressed in astrocytes. Additionally, gastrodin contributed to the restoration of the blood–brain barrier (BBB) and modulated the expression levels of inflammatory mediators interleukin‐6 (IL‐6), tumor necrosis factor‐alpha (TNF‐α), and matrix metallopeptidase 8 (MMP8).

**Conclusion:**

Gastrodin treatment has the potential to mitigate tau pathology, thereby enhancing learning and memory in AD mouse models. This effect may be mediated through the modulation of cerebral mechanical viscoelasticity via the mechanosensor FERMT2, which facilitates the restoration of synaptic structure and function. This process is potentially linked to the maintenance of BBB integrity and the modulation of inflammatory factor release.

## Graphical abstract

Gastrodin reduces tau hyperphosphorylation in 3xTg‐AD mice, reverses decreased brain matrix viscoelasticity, and remodels ECM components by targeting the Alzheimer's risk gene FERMT2, primarily in astrocytes. It binds to FERMT2, inhibiting its expression, which leads to ECM remodeling, restored matrix viscoelasticity, and blood–brain barrier recovery. Gastrodin also modifies neuroinflammatory factors such as TNF‐α, IL‐6, and MMP8.

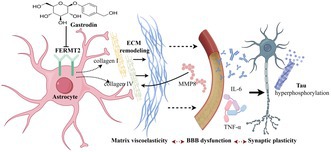

## 1 Introduction

Alzheimer's disease (AD) is a progressive neurodegenerative disorder marked by a gradual deterioration in cognitive function, with its prevalence rising in tandem with the aging population [1]. Despite substantial research efforts, effective prevention and treatment strategies have yet to be realized [1]. The etiology of AD is complex, involving a significant asymptomatic phase that can persist for approximately 20 years prior to the manifestation of cognitive impairments. Recent research has demonstrated that the viscoelastic properties of brain tissue markedly diminish with advancing age and the progression of AD, a phenomenon corroborated by magnetic resonance elastography [2]. Alterations in brain viscoelasticity are linked to various mediating factors, notably within the burgeoning field of mechanosensors [3]. These mechanosensors have the capacity to detect variations in the physical characteristics of the extracellular matrix (ECM), such as viscoelasticity and stiffness, and transduce these mechanical signals into biochemical signals [4, 5, 6]. This process subsequently regulates cellular behavior through a cascade of downstream signaling pathways. Despite advancements in the field, research on the alterations in the physical properties of the brain and the underlying mechanisms of sensory action remains insufficient. Furthermore, changes in the viscoelasticity of the ECM may enhance the permeability of the blood–brain barrier (BBB), thereby undermining the brain's protective mechanisms [7, 8, 9]. Consequently, it is essential to elucidate the relationship between the reduction in brain viscoelasticity and the integrity of the BBB [10, 11]. Current endeavors aimed at reversing the deterioration of brain viscoelastic properties and clarifying their implications for BBB function, particularly in the context of AD, necessitate further investigation. A more comprehensive understanding of these mechanisms could facilitate the development of novel therapeutic strategies to address cognitive decline in patients with AD.

Gastrodin, a bioactive compound derived from Gastrodia elata, has garnered attention as a promising therapeutic agent for neurodegenerative diseases because of its diverse and multi‐faceted mechanisms of action [12, 13, 14]. These include neuroprotective effects, modulation of neurotransmitter systems, anti‐inflammatory and antioxidant activities, inhibition of microglial activation, and regulation of mitochondrial pathways [15, 16, 17]. In cellular and animal models of AD, gastrodin exhibits multiple mechanisms of action, including the inhibition of BACE1 expression, reduction of tau hyperphosphorylation, and prevention of amyloid‐beta (Aβ) aggregation [12, 18]. It also mitigates Aβ‐induced cytotoxicity and ameliorates cognitive impairments in animal studies. Furthermore, research indicates that gastrodin attenuates neuroinflammatory responses by modulating signaling pathways such as NF‐κB and MAPK, thereby safeguarding neurons and enhancing neural function [14, 19, 20]. This multi‐targeted approach underscores its promise as a therapeutic candidate for addressing not only neurodegenerative diseases but also neural injuries. Recent studies highlight gastrodin's substantial influence on the BBB, where it enhances barrier integrity and decreases permeability through diverse mechanisms, thereby safeguarding brain tissue from deleterious substances [21, 22]. Furthermore, alterations in brain viscoelasticity may impact the interactions between neurons and glial cells, consequently affecting the integrity and functionality of the BBB [23, 24]. Investigating viscoelasticity offers a novel perspective on cellular responses to gastrodin treatment. Nonetheless, the specific mechanisms through which gastrodin mitigates the reduction in viscoelasticity of the brain's ECM to improve BBB integrity require further elucidation.

The integrin‐associated receptor FERMT2 is posited to play a pivotal role in mediating the interactions between brain viscoelasticity, ECM dynamics, and BBB functionality, thereby significantly impacting the progression of AD [25, 26]. Members of the Kindlin family, which are essential for integrin activation, have been recognized as critical transmembrane adhesion receptors. Notably, Kindlin‐2 (FERMT2) has been identified as a risk gene for AD through numerous genome‐wide association studies (GWAS) and polygenic risk scores (PRS) [27, 28]. Significantly, single nucleotide polymorphisms (SNPs) of FERMT2 are implicated in both familial Alzheimer's disease (fAD) and late‐onset Alzheimer's disease (LOAD), demonstrating the most pronounced association with stages of mild cognitive impairment [29, 30]. FERMT2 is broadly present in the ECM and mitochondria of fibroblasts, cardiomyocytes, vascular endothelial, and tumor cells, serving as a bidirectional link between integrin and actin [30, 31, 32, 33]. As an innovative mechanosensor, FERMT2 relocates to the nucleus and mitochondria in response to alterations in the physical microenvironment of the cell, thereby augmenting integrin activation. Within the mitochondria, FERMT2 engages in interaction with the proline‐synthesizing enzyme pyrroline‐5‐carboxylate reductase 1 (PYCR1), resulting in elevated proline concentrations in cells exposed to modified physical conditions, such as rigid ECMs [32]. This mechanosensitive pathway has been substantiated in advanced‐stage lung adenocarcinoma. Our initial investigations have demonstrated that the viscoelastic properties of brain tissue in 3xTg‐AD model mice deteriorate as the disease progresses, suggesting that this risk gene is involved in mechanosensing and ECM remodeling. Further research is necessary to determine whether modulation of FERMT2 can reverse the decline in brain ECM viscoelasticity and evaluate its potential as a therapeutic target in AD interventions.

Research has demonstrated that gastrodin facilitates the synthesis and remodeling of ECM components, although the precise mechanisms underlying these effects have yet to be fully elucidated [34]. Recent investigations propose that gastrodin augments the proliferation and migration of fibroblasts, which are integral to the processes of ECM synthesis and remodeling [35, 36]. Additionally, gastrodin has been identified as a regulator of critical cytokines involved in ECM remodeling, including transforming growth factor‐beta (TGF‐β) and matrix metalloproteinases (MMPs), both of which play essential roles in ECM degradation and regeneration [37, 38]. Additionally, certain studies indicate that gastrodin can enhance the expression of ECM components such as collagen and hyaluronic acid, thereby promoting tissue repair and regeneration. In neural injury models, gastrodin has been shown to support neuronal survival and facilitate ECM remodeling, thereby contributing to neural repair [39, 40]. The potential of gastrodin to counteract the reduction in brain ECM viscoelasticity through the modulation of mechanosensing factors, particularly FERMT2, and its implications for the restoration of BBB functionality and the mitigation of neuroinflammation, necessitates further investigation. Elucidating these mechanisms may facilitate the development of novel therapeutic strategies aimed at targeting ECM dynamics in AD.

## 2 Method and Animals

### 2.1 Materials, Lab Animals, Reagents, and Antibodies

Experimental animals were maintained in a sterile environment at Guizhou Medical University, with controlled temperature, 12‐h light/dark cycles, and humidity. DNA was extracted from neonatal mice for genotype identification using PCR and electrophoresis. Previous studies showed that gastrodin improved cognitive dysfunction in AD mouse models dose‐dependently. In mice with vascular dementia, gastrodin at 22.5 and 90 mg/kg effectively enhanced cognitive function, with the 90 mg/kg dose being more effective [41]. In a D‐galactose‐induced AD model study, the 90 mg/kg gastrodin treatment improved cognitive dysfunction in AD mice more effectively than the 210 mg/kg treatment [42]. In LPS‐induced mouse models of cognitive function, the 100 mg/kg gastrodin group showed effects comparable to the donepezil group [14]. Thus, we used two gastrodin doses, 50 and 100 mg/kg, to assess its impact on cognitive dysfunction in 3xTg‐AD mice. After 30 days of intragastric administration of low (50 mg/kg) and high (100 mg/kg) doses of gastrodin to 3xTg‐AD mice, cognitive function assessments and related experimental studies were subsequently conducted. A detailed list of materials, including chemicals, antibodies, and kits, is provided in Tables S1 and S2.

### 2.2 Morris Water Maze Test

The Morris water maze (MWM) tested mice's cognitive abilities using a 140 cm diameter, 50 cm high circular pool filled with opaque white water at 23°C–25°C, divided into four quadrants. After 4 days of training, the platform was removed, and mice swam freely for 60 s. Trials were recorded and analyzed using the EthoVisionXT system by Noldus Information Technology. Key metrics were time to reach the platform in learning trials, platform crossings count, time to first cross the platform location, and speed in the probe trial.

### 2.3 Y‐Maze Spontaneous Alternate Test

The Y‐maze consists of three equally sized arms arranged at 120° angles, forming an equilateral radial pattern, made of opaque, acid‐resistant material. Mice are placed with their back to the central area and allowed to explore the arms freely for up to 5 min. The maze is cleaned with alcohol‐soaked cotton to remove scent traces between trials. The total number of entries and entry sequences are recorded using a shadow image acquisition system. A mouse is considered to have explored an arm successfully when all four limbs enter, and the movement within the arm is tracked. A correct alternation is defined as alternating between the three arms without repetition, recorded as one correct alternation. The total number of arm entries within the 5‐min period reflects the mouse's spatial exploration ability. The spontaneous alternation frequency is calculated as: (Correct alternation count/Total arm entries − 2) × 100%, assessing spatial recognition and memory.

### 2.4 Western Blot

Mouse brain tissues are lysed with RIPA buffer and inhibitors, chilled on ice for 30 min, and sonicated. Protein levels are determined using the BCA method. Samples are mixed with loading buffer, boiled for 5 min, and run on SDS‐PAGE. Proteins are transferred to a PVDF membrane, blocked with 5% skim milk for 2 h at room temperature, and incubated overnight at 4°C with primary antibodies. After washing with TBST buffer, HRP‐conjugated secondary antibodies are added and incubated for 1 h at room temperature. Detection is done using ECL chemiluminescence, followed by image capture. The list of antibodies used in the Western blot experiments is provided in Table S1.

### 2.5 Atomic Force Microscopy

Mouse brain tissue slices, measuring 100 μm in thickness, were affixed to a scaffold and subsequently immersed in artificial cerebrospinal fluid. The atomic force microscopy (AFM) scanning parameters were optimized to ensure optimal contact between the sample and the probe. The scanning program facilitated the probe's movement to accurately capture the topographical features of the sample. In this study, the Young's modulus index was employed to analyze and model the needle insertion curve. Here, E1 denotes the Young's modulus of the sample, “Tip Geometry” refers to the fitting model of the probe, and “Radius” indicates the radius of the probe utilized in the fitting process for determining the Young's modulus.

### 2.6 Silver Staining

Sections were sequentially immersed in Xylene I and II for 20 min each, followed by anhydrous ethanol I and II for 5 min each, and 75% ethanol for 5 min. They were then washed with tap water and rinsed with distilled water until clean. The sections were stained with Glycine Silver Staining Solution C for 5 min, rinsed three times with distilled water, and treated with Solution B for 3–5 min. The sections were flicked to remove excess Glycine Silver Solution B, then briefly placed in Glycine Silver Staining Solutions A I and A II, rinsed with distilled water, and sequentially immersed in anhydrous ethanol I, II, and III, followed by Xylene I and II, each for 5 min.

### 2.7 Golgi Staining

Mouse brain tissue was extracted, rinsed with water, and soaked in solutions A and B for 2 weeks. It was then moved to liquid C for 72 h. Refresh the immersion solution after 6 h or the following day. After 2 weeks, transfer to liquid C for another 72 h, replacing the liquid the next day. Cut tissue into 100 μm slices using a cryotome. Rinse slices with water three times for 4 min each. Soak in a mixture of solutions D and E with water for 10 min. Rinse with Milli‐Q or distilled water three times for 4 min each. Restain with crystal violet, dehydrate using ethanol gradients, and seal with xylene and neutral resin.

### 2.8 Transmission Electron Microscope

Mice were anesthetized and perfused with fixative, after which hippocampal tissue was extracted and sectioned into 1 mm^3^ blocks. These blocks were fixed with glutaraldehyde, rinsed with phosphate buffer, and postfixed in osmium tetroxide. Following dehydration in ethanol and acetone, the samples were infiltrated with acetone and embedding medium, then polymerized at 37°C, 45°C, and 60°C. Ultrathin sections (70 nm) were cut using an ultramicrotome. The sections were stained with a 2% saturated aqueous solution of uranyl acetate in darkness. Subsequently, the samples were examined using a transmission electron microscope (HITACHI, HT7800/HT7700) for acquiring and analyzing ultrastructural images of the BBB within the hippocampal tissue.

### 2.9 Immunofluorescence Staining of Frozen Section

Frozen slices of mice from different treatment groups were thawed at room temperature for 30 min, washed three times in 1 × PBS for 10 min each, and fixed in 4% paraformaldehyde for 30 min. The tissue was then incubated in 1 × PBST for 20 min and blocked with goat serum for 1 h. Primary antibody solution was applied, and the sections were incubated overnight at 4°C. After washing with pre‐cooled 1 × PBS, a fluorescent secondary antibody was added and incubated for 1 h at room temperature. Following a final rinse with 1 × PBS, the samples were mounted with DAPI tablets.

### 2.10 Immunohistochemistry

Paraffin sections were preheated at 56°C for 1 h, dewaxed in xylene, and rehydrated with graded ethanol. Antigen retrieval used hot citrate buffer, followed by 3% H_2_O_2_ to block peroxidase. Sections were incubated in goat serum for 1 h, then with primary antibody overnight at 4°C. The next day, fluorescent secondary antibody was applied for 1 h at room temperature. Color development was done using DAB solution, followed by a 10‐s hematoxylin stain. Sections were then dehydrated, cleared with xylene, and sealed with neutral gum for imaging and analysis.

### 2.11 Molecular Docking

The receptor structure preparation for docking entailed obtaining the three‐dimensional configuration of the protein FERMT2 (PDB ID: 5XPZ) from the Protein Data Bank (PDB), which served as the basis for this investigation. For the ligand preparation, the molecular structure of gastrodin was initially constructed using ChemBio 2D Ultra 14.0 and subsequently converted into PDB file format via PyMOL. The ligand underwent energy minimization using ChemBio 3D Ultra 14.0 in accordance with established protocols. Ultimately, the molecular docking of gastrodin with the 5XPZ structure was conducted utilizing AutoDock Vina. The interactions between gastrodin and 5XPZ were examined utilizing Discovery Studio 2016 Client and PyMOL software.

### 2.12 Cellular Thermal Shift Assay

Mouse brain tissue was homogenized in RIPA lysis buffer with a protein homogenizer for protein extraction. The homogenate was divided equally into eight PCR tubes (200 μL each). Thermal denaturation was performed using a gradient PCR machine, with temperatures ranging from 35°C to 75°C for 5 min. The samples then underwent three freeze–thaw cycles using liquid nitrogen. After centrifuging at 4°C and 20,000 × *g* for 20 min, the supernatant was collected. A 5× loading buffer was added, and the mixture was boiled for 5 min to inactivate enzymes. A 10 μL sample was then analyzed by Western blotting with SDS‐PAGE.

### 2.13 Statistical Analysis

Experimental data were shown as (Mean ± SD). GraphPad Prism 9 handled all statistical analyses and visualizations, with *p* < 0.05 indicating significance. The analysis begins with a normality test (e.g., the Kolmogorov–Smirnov test). If the data follow a normal distribution, One‐Way ANOVA is applied for group comparisons, and an independent sample *t*‐test is used for comparisons between two groups. If the data does not exhibit a normal/Gaussian distribution, non‐parametric equivalent tests should be used for analysis. The statistical method used in the Golgi stain analysis is as follows.

Statistical analysis of the Number of Intersections was evaluated by Sholl analysis. Using the Sholl analysis plug‐in in ImageJ software, equal‐spaced concentric circles (spacing set to 10 μm) were drawn outward from the center of the cell body, and the number of junctions between the dendrites and each circle was counted. The results were drawn as a curve of the relationship between the Number of Intersections and the distance, and the average number of intersections of each experimental group was recorded as an evaluation index. Data are expressed as mean ± SD.

Nested analysis is used in the statistical examination of Spine Density in Golgi staining. Randomly select 5–10 stained and intact neurons from each group and analyze their secondary or tertiary dendritic segments. Collect high‐resolution images using a light microscope (100× oil objective). Use ImageJ software to mark the 2D projection area of the dendrites and calculate the area (μm^2^). Count all dendritic spines within the marked dendritic segments in the microscopic images, and calculate the spine density per unit area using the following formula:

$$\begin{array}{c} \text{Spine density}\;\mathrm{per}\;100\;{\mathrm{\mu m}}^{2}\\ \;=\left[\text{Total number of spines}/\text{Projection area of dendrites}\;\left({\mathrm{\mu m}}^{2}\right)\right]\times 100 \end{array}$$

In the statistical analysis of mushroom (%) in Golgi staining, nested analysis is applied. Each group randomly selects at least 5 neurons, with 3 dendritic segments (20–30 μm in length) from secondary or tertiary dendrites analyzed per neuron. Dendritic spine morphology (mushroom, thin, and stubby) is observed and classified using a light microscope (100× oil objective). The number of Mushroom‐type spines and the total number of spines on the dendritic segments are counted using ImageJ software. The calculation formula for Mushroom (%) is:

$$\begin{array}{c} \text{Mushroom}\;\left(\%\right)=[\text{The number of mushroom}-\text{type dendritic spines}\\ \;/\text{Total number of dendritic spines}]\times 100. \end{array}$$

## 3 Results

### 3.1 Gastrodin Alleviates Learning, Memory, and Spatial Recognition Abilities in 3xTg‐AD Mice

**FIGURE 1:**
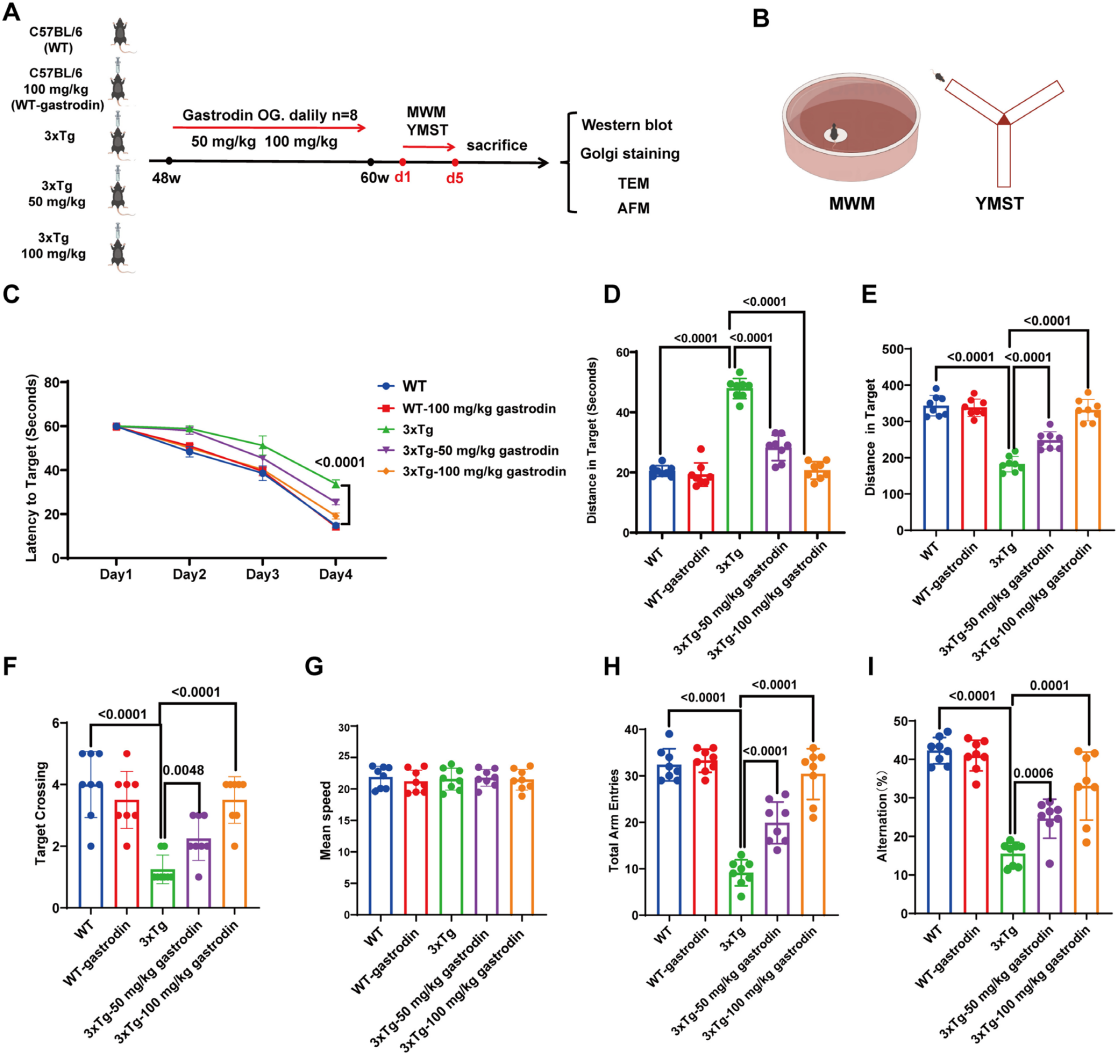
Gastrodin ameliorated cognitive deficits in 3xTg‐AD mice. (A) A schematic diagram illustrating the gastrogastroplication procedure in AD mice, administered with varying doses of gastrodine, along with an assessment of behavioral changes and key detection parameters posttreatment. In this context, “48w” and “60w” denote the equivalent durations of 48 weeks and 60 weeks in terms of mouse months, respectively. (B) Schematics of behavioral tests such as Morris Water Maze and Y‐Maze. (C) The latency to locate the platform was recorded from days 1 to 4. (D–G) Analysis of differences in escape latency, target quadrant distance, target quadrant residence time, distance traveled, crossing of the target quadrant, and average speed in the Morris Water Maze; (H, I) Assessment of the total number of entries into arms and the correct rate of arm entries in the Y‐Maze spontaneous alternation experiment (*n* = 8 independent experiments). Significant *p* values are indicated on the graph. Two‐way ANOVA and Tukey's multiple comparison test were used.

In this study, following a 30‐day regimen of gastrodin administration via gavage at low (50 mg/kg) and high (100 mg/kg) doses in AD mice, it was observed that both dosage groups exhibited a reduced escape latency relative to the untreated AD group (Figure 1C). Additionally, there was an increase in the time spent in the target quadrant (Figure 1D), the distance traveled, and the frequency of crossings into the target quadrant (Figure 1E,F). However, no significant differences were detected in the average speed across all groups of mice (Figure 1G). The findings from the Y‐maze spontaneous alternation test revealed that both low and high doses administered to AD mice resulted in a significant increase in total entries and correct entry rates relative to the control group (Figure 1H,I). These results suggest that both dosage levels substantially enhance cognitive function and spatial recognition abilities in AD mice. Furthermore, the administration of a high dose does not adversely affect cognitive function in wild‐type mice.

### 3.2 Gastrodin Mitigates the Formation of Neurofibrillary Tangles and Diminishes the Hyperphosphorylation of Tau Protein

**FIGURE 2:**
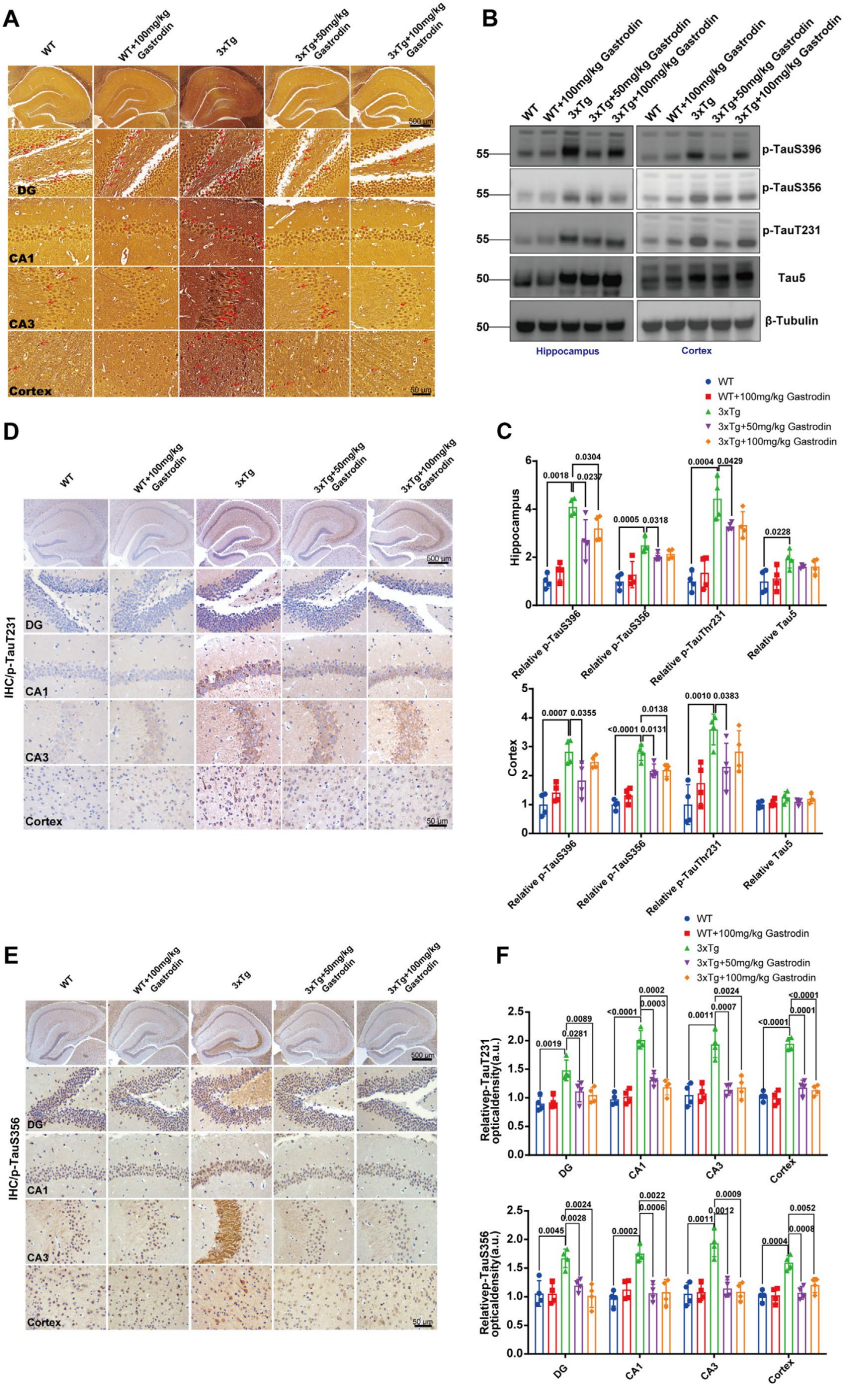
Gastrodin reduces phosphorylated Tau protein expression in the brains of 3xTg‐AD mice. (A) Representative images of neurofibrillary tangles in mice from each experimental group. The DG (dentate gyrus), CA1, and CA3 regions of the hippocampus are indicated in the figure; (B, C) Representative blots and quantification of p‐Tau S396, p‐Tau S356, p‐Tau T231 and Tau5 in hippocampus and cortex of mice in each group (*n* = 8 independent experiments); (D, F) Immunohistochemical representative images and quantification of Tau phosphorylation at site S356 in neurons of mice were obtained for each group (*n* = 8 independent experiments); (E, F) Immunohistochemical representative images and quantification of Tau phosphorylation at site T231 in neuronal cells of mice within each experimental group (*n* = 8 independent experiments); Significant *p* values are indicated on the graph. One‐way ANOVA and Tukey's multiple comparison test were used.

Silver staining analysis indicated a notable reduction in the formation of neurofibrillary tangles in AD mice treated with the drug, in comparison to the AD control group (Figure 2A). Furthermore, Western blot analysis of hippocampal and cortical tissues across all experimental groups revealed that oral administration of gastrodin at low (50 mg/kg) and high (100 mg/kg) doses resulted in a significant decrease in the protein levels of phosphorylated tau at sites p‐S396, p‐S356, and Thr231, relative to the AD control group, in both the hippocampus and cortex (Figure 2B,C). In the Western blot analysis of the hippocampus, the reduction of tau S356 in the low‐dose group (50 mg/kg) was statistically significant. However, for tau Thr231, the decrease in both the hippocampus and cortex in the 50 mg/kg group was statistically significant. Immunohistochemistry (IHC) results demonstrated a marked reduction in the expression levels of p‐S356 (Figure 2D,F) and p‐Thr231 (Figure 2E,F) subsequent to gastrodin treatment. This suggests that gastrodin administration via gavage effectively mitigates the pathological process associated with excessive phosphorylation of tau protein in 3xTg‐AD mice. Importantly, no significant differences in tau‐related indicators were detected in wild‐type mice administered high doses of gastrodin compared to untreated wild‐type controls.

### 3.3 Gastrodin Ameliorates Synaptic Impairment in Murine Models of Alzheimer's Disease

**FIGURE 3:**
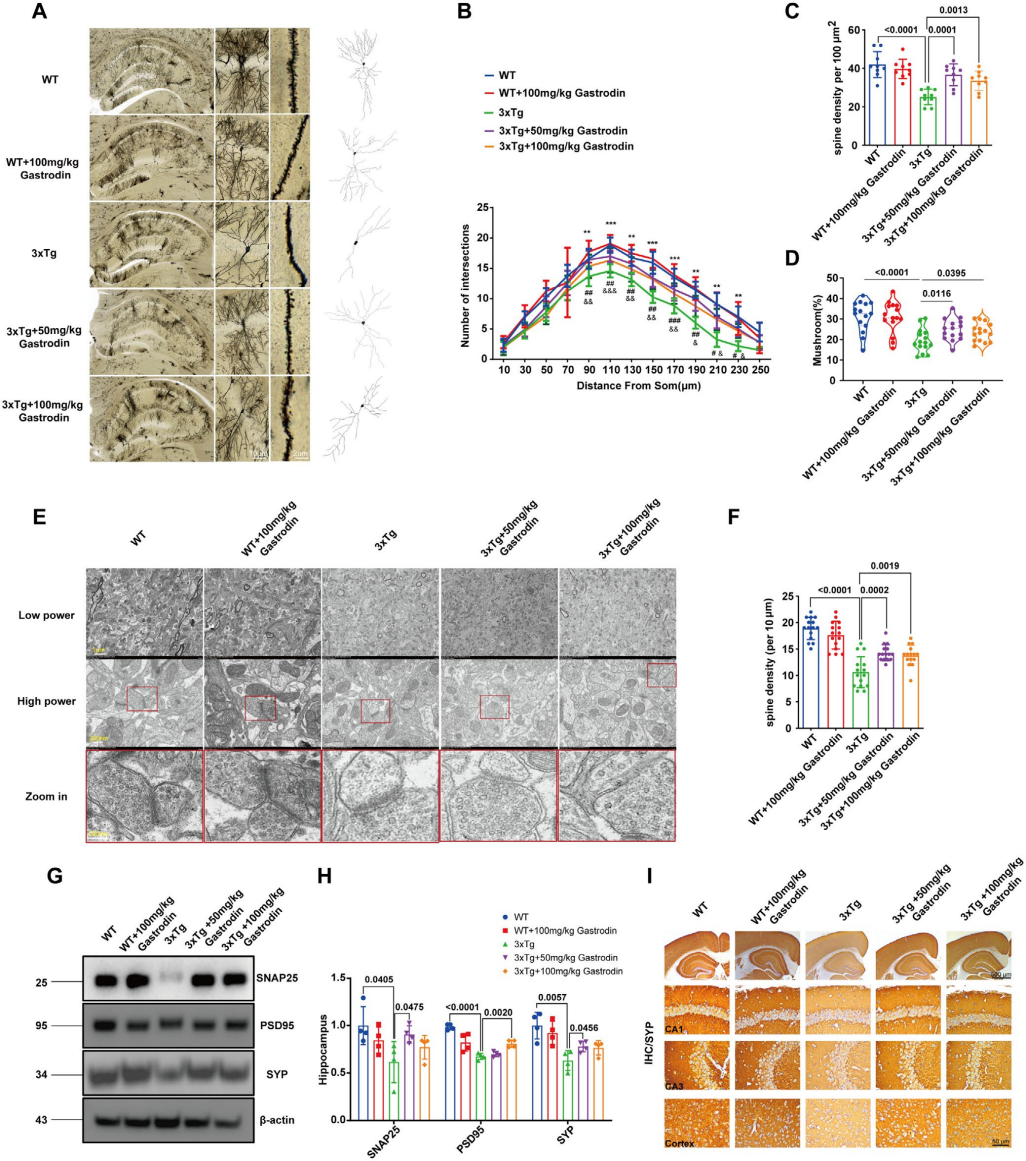
Gastrodin improves synaptic damage in 3xTg‐AD mice. (A) Golgi stain images of mice in each group; (B) Statistics on axon complexity, **p < 0.001, ***p < 0.0001 (the WT group vs. the 3xTg group); ^#^ *p* < 0.05, ^##^ *p* < 0.001, ^###^ *p* < 0.0001 (the 3xTg group vs. the 3xTg + 100 mg/kg Gastrodin group), ^&^ *p* < 0.05, ^&&^ *p* < 0.001, ^&&&^ *p* < 0.0001 (the WT group vs. the 3xTg + 50 mg/kg Gastrodin group); (C) Statistics on the density of dendritic spines; (D) Statistics on mushroom‐shaped dendritic spines; (E) Example of synaptic transmission observed using electron microscopy; (F) Statistics on synaptic vesicles; (G, H) Representative blots and quantification of synaptic proteins in the hippocampus and cortex of mice in all groups; (I) Representative immunohistochemical images illustrating Synaptophysin (SYP) expression. Significant *p* values are indicated on the graph. One‐way ANOVA, two‐way ANOVA and Tukey's multiple comparison test were used.

Golgi staining of the experimental mice demonstrated a significant increase in axonal complexity and dendritic spine density in hippocampal neurons of AD mice treated with both low and high doses of gastrodin, compared to the control AD group (Figure 3A–D). Mushroom‐shaped dendritic spines are a distinct subtype of dendritic spines characterized by a bulbous head and a narrow neck, which are considered indicative of mature synaptic connections. These spines are highly enriched in postsynaptic density proteins and neurotransmitter receptors, making them critical for stable and efficient synaptic transmission. In the context of our results, the prevalence or alterations in mushroom‐shaped spines suggest changes in synaptic stability and plasticity, which are essential for processes such as learning and memory. Our analysis of Figure 3D reveals that gastrogastrotherapy with gastrodin resulted in an increase in the density or proportion of mushroom‐shaped dendritic spines. This finding implies that enhanced synaptic stabilization. These morphological changes highlight the potential impact of gastrodin on synaptic architecture and function, which we further discuss in relation to its implications for neuronal health and connectivity in the Discussion section. Additionally, transmission electron microscopy analysis revealed a substantial increase in the number of synaptic vesicles in the low and high dose gastrodin‐treated AD mice relative to the AD control group (Figure 3E,F). Furthermore, western blot analysis revealed that the expression levels of the presynaptic proteins SNAP25 and SYP were elevated in the low‐dose AD mice relative to the AD control group. In contrast, the expression of the postsynaptic protein PSD95 was significantly enhanced in the high‐dose AD mice (Figure 3G,H). Immunohistochemical analysis corroborated a consistent pattern of SYP expression across the hippocampal CA1 and CA3 regions, as well as the cortical area (Figure 3I). Importantly, no statistically significant differences in these expression levels were detected between wild‐type mice administered high doses of gastrodin and untreated wild‐type control mice.

### 3.4 Gastrodin Has the Potential to Counteract the Reduction in Matrix Viscoelasticity by Modulating Extracellular Matrix Components, Specifically Collagen Types I and IV

**FIGURE 4:**
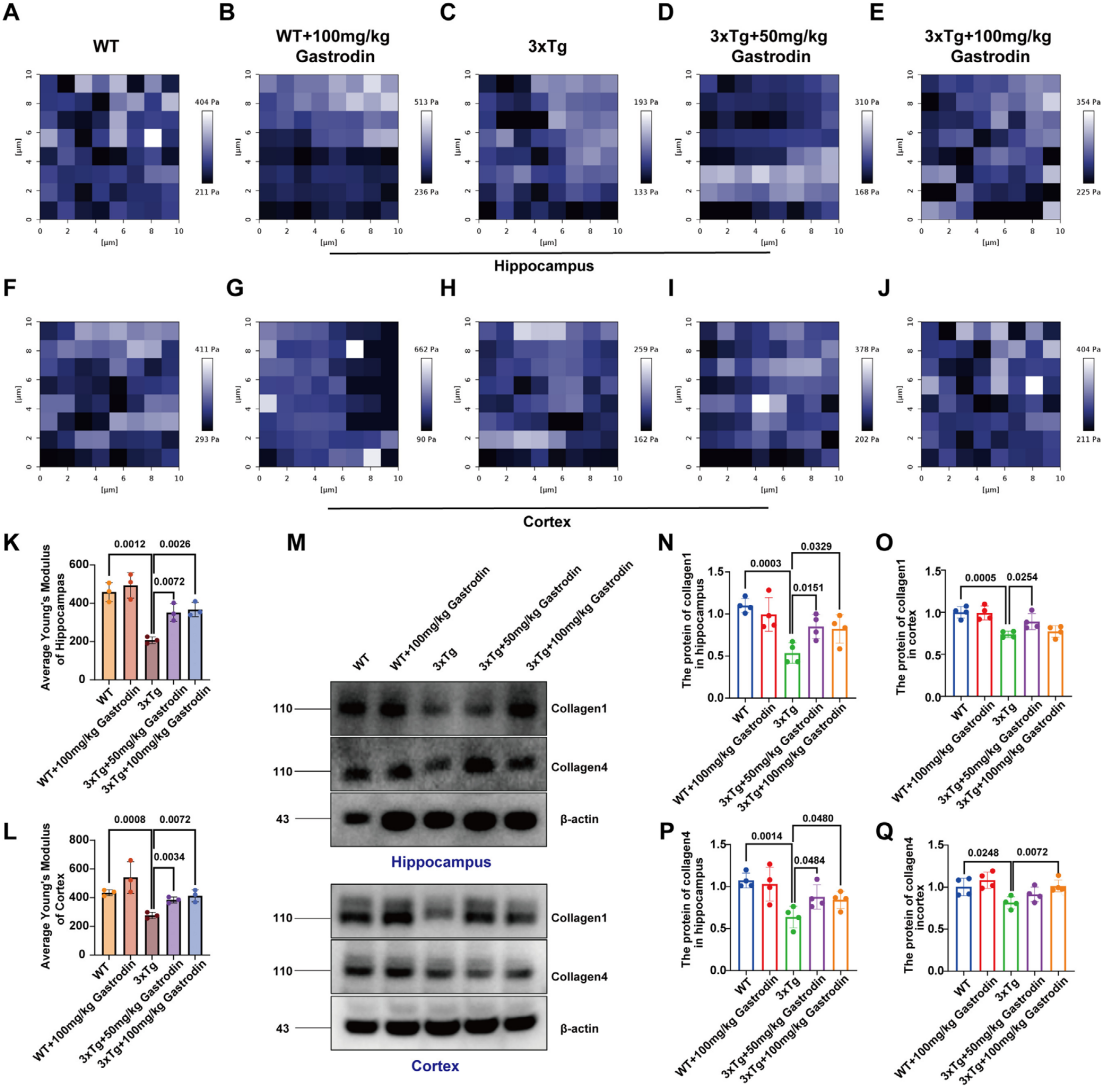
Gastrodin reverses the decrease in mechanical viscoelasticity of the brain by affecting the remodeling of extracellular matrix (collagen 1, collagen 4). (A–J) AFM imaging of hippocampus in the hippocampus and cortex of mice in all groups; (K) Comparison of Young's modulus in hippocampal area of mice in each group; (L) Comparison of Young's modulus in cortical region of mice in each group; (M–Q) Representative blots and quantification of collagen 1 and collagen 4 in hippocampus and cortex of mice in each group (*n* = 8 independent experiments); Significant *p* values are indicated on the graph. One‐way ANOVA and Tukey's multiple comparison test were used.

Previous studies have demonstrated that the Young's modulus, measured using AFM, serves as a reliable quantitative parameter for representing the viscoelastic properties of tissue matrices [43, 44]. In this study, AFM analysis of brain tissue from each group of mice revealed an increase in the mean Young's modulus in both the hippocampal and cortical regions of mice treated with low and high doses of gastrodine compared to the 3xTg group (refer to Figure 4A–L). Subsequent Western blot analyses revealed a significant upregulation in the expression of ECM components, specifically collagen types I and IV, within the hippocampal and cortical regions of AD model mice administered both low and high doses of gastrodin, in comparison to the control AD group (Figure 4M–Q). In contrast, no significant differences were detected in wild‐type mice treated with high doses of gastrodin relative to untreated wild‐type controls. These findings suggest that gastrodin may counteract the reduction in matrix viscoelasticity by modulating ECM components.

### 3.5 Gastrodin May Modulate Matrix Viscoelasticity Through the Inhibition of Mechanosensor FERMT2 Expression in Astrocytes

**FIGURE 5:**
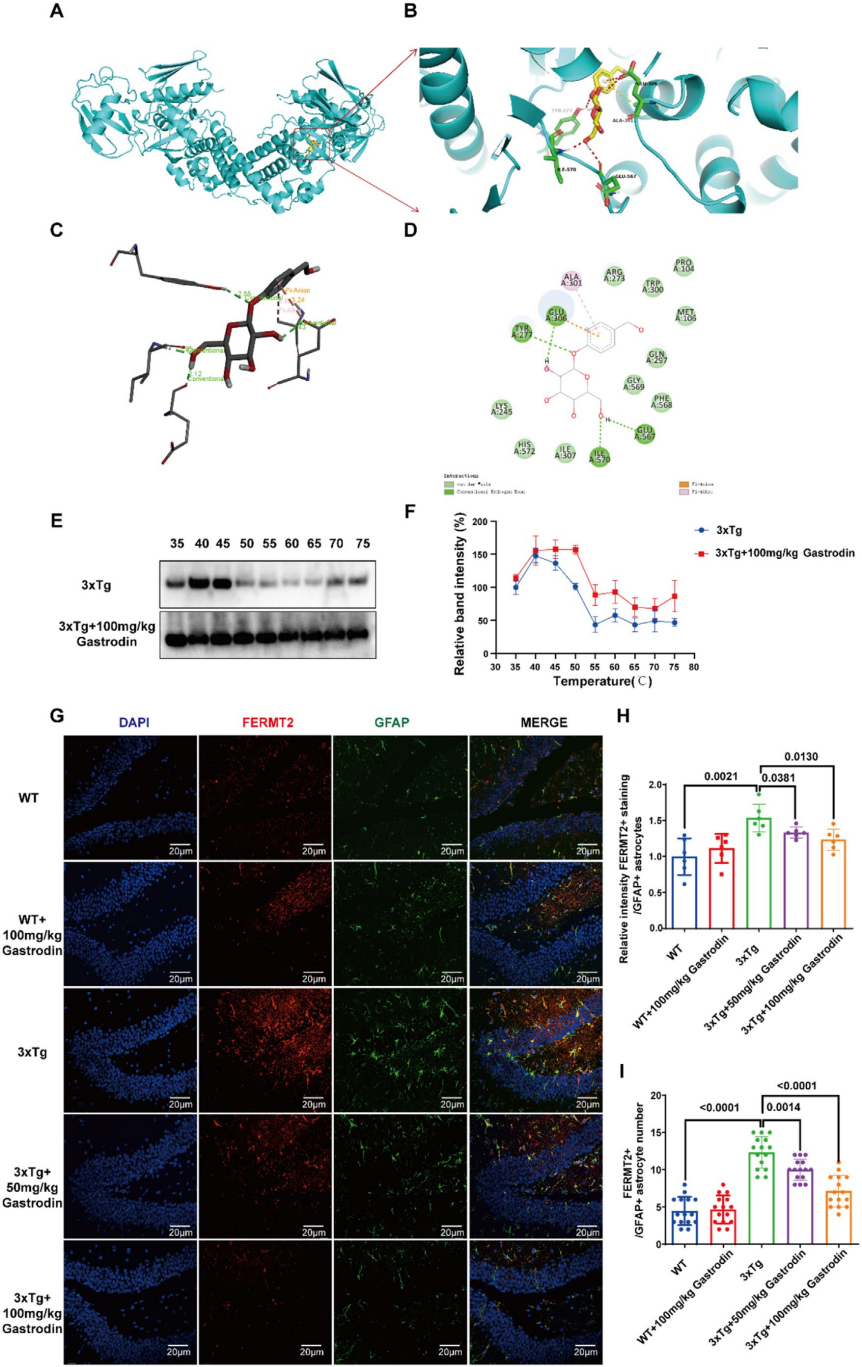
Gastrodin may affect matrix viscoelasticity by inhibiting the expression of mechanical sensing factor FERMT2 in astrocytes. (A–C) Three‐dimensional representations of the molecular docking interactions between gastrodin and FERMT2. (D) Two‐dimensional representations of the molecular docking interactions between gastrodin and FERMT2. (E) Representative Western blot images illustrating FERMT2 expression in CETSA results. (F) Quantitative assessment of the melting curve for FERMT2 in CETSA. (G) Representative imprinting of FERMT2 in hippocampus of mice in all groups (*n* = 4 independent experiments). (H, I) FERMT2 fluorescence intensity of astrocytes in hippocampus of mice in all groups and the number of positive cells colocated with GFAP (*n* = 4 independent experiments). Significant *p* values are indicated on the graph. One‐way ANOVA, Tukey's multiple comparison test.

Expanding upon our prior research, this study sought to ascertain whether the mechanosensor FERMT2, predominantly expressed in astrocytes, could serve as a potential target for gastrodin in modulating matrix viscoelasticity. Molecular docking analysis conducted with AutoDock Vina demonstrated a binding energy of −6.8 kcal/mol between gastrodin and FERMT2, suggesting a strong binding affinity (refer to Figure 5A–D). Moreover, the Cellular Thermal Shift Assay (CETSA) revealed a progressive reduction in the intensity of the specific band corresponding to FERMT2 with increasing temperature. This observation implies that gastrodin stabilizes the target protein, as evidenced by a notable rightward shift in the melting curve posttreatment. Such a shift corroborates the capacity of gastrodin to bind directly to FERMT2 (Figure 5E,F). In our previous studies, we established that FERMT2 is predominantly expressed in astrocytes (refer to Supporting Information). Additionally, immunofluorescence analysis demonstrated a significant reduction in the fluorescence intensity of FERMT2 in astrocytes, as well as a decrease in the number of GFAP colocalized positive cells in 3xTg mice following gastrodin treatment (Figure 5G–I). These findings substantiate the inhibitory effect of gastrodin on the expression of the mechanosensor FERMT2 in astrocytes.

### 3.6 Gastrodin Mitigates the Disruption of the BBB in 3xTg‐AD Mice

**FIGURE 6:**
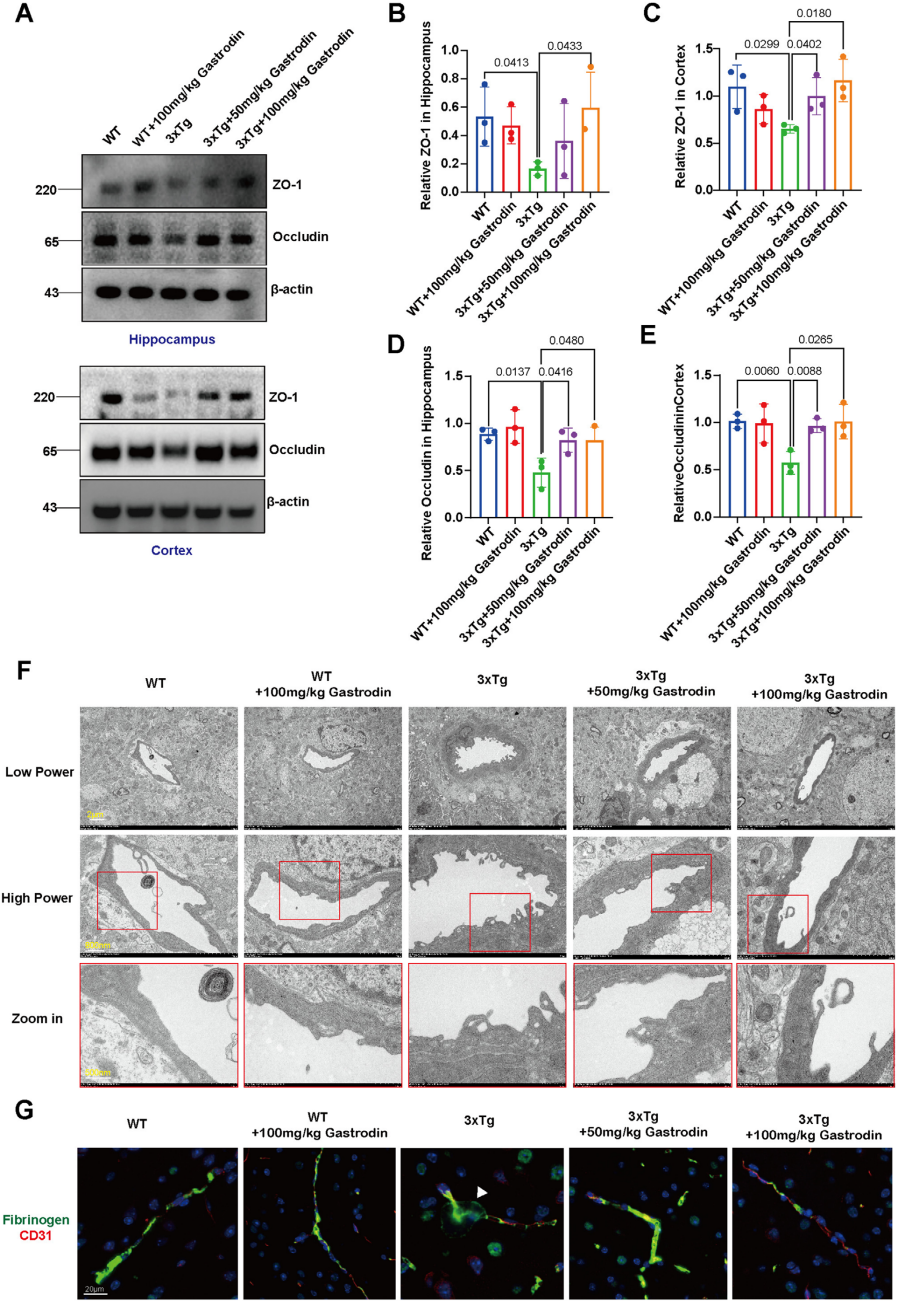
Gastrodin improves BBB structure and function in the brains of 3xTg‐AD mice. (A) Representative blots of occludin and ZO‐1 protein expression levels in the mouse hippocampus and cortex. (B–E) Quantification analysis of occludin and ZO‐1 protein expression. Significant *p* values are indicated on the graph. One‐way ANOVA and Tukey's multiple comparison test were used, *n* = 3. (F) Representative transmission electron microscopy images of the BBB in the hippocampal region of each group of mice. (G) Representative dual‐immunofluorescence staining image of Fibrinogen (green) and CD31 (red), with a scale bar of 20 μm.

To investigate the impact of gastrodin on BBB functionality in AD mouse models, western blot analysis was employed to quantify the expression levels of gap junction and tight junction proteins, with a focus on Occludin and ZO‐1. The findings demonstrated a significant increase in the expression of Occludin and ZO‐1 in the hippocampal and cortical regions of AD mice administered with both low and high doses of gastrodin, in comparison to the control AD group (Figure 6B–F). In the analysis of the hippocampus, the difference in ZO‐1 levels in the low‐dose group (50 mg/kg) was statistically significant. No significant differences were observed between high‐dose gastrodin‐treated wild‐type mice and untreated wild‐type controls. Furthermore, transmission electron microscopy demonstrated that the endothelial cells and basement membranes in AD mice treated with both low and high doses of gastrodin appeared smoother and more intact compared to those in the control AD group (Figure 6F). As for the observation of smoother and more intact vessels after gastrodin treatment, we hypothesize that the treatment may enhance endothelial cell stability and reduce cellular disruption, which could lead to a more uniform appearance of the vessel walls. This effect might be associated with the potential protective and restorative properties of gastrodin on the endothelial barrier function, thereby improving the integrity of the BBB in AD mice. Further investigations are required to fully elucidate the underlying mechanisms. Fibrinogen was used as a marker of BBB leakage. In both wild‐type controls and Gastrodin‐treated 3xTg‐AD mice, fibrinogen was detected exclusively within the vascular lumen, indicating an intact BBB (Figure 6G). However, in 3xTg‐AD mice, a significant extravascular accumulation of fibrinogen was observed in the brain parenchyma (Figure 6G), supporting the notion that Gastrodin can enhance the structural integrity of the BBB.

### 3.7 Gastrodin Modulates Neuroimmune Responses in 3xTg‐AD Mice

**FIGURE 7:**
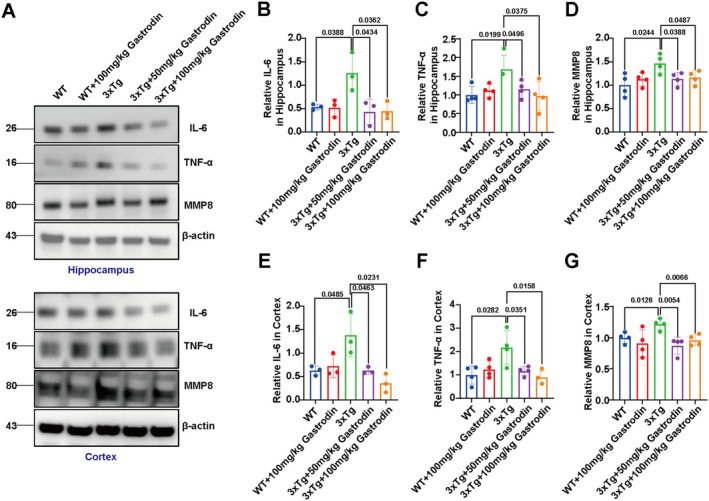
Gastrodin alleviates neuroinflammation in 3xTg‐AD mice. (A) Representative blots of IL‐6, TNF‐αand MMP8 protein expression levels in the mouse hippocampus and cortex. (B) The quantification of IL‐6 in hippocampus of mice in each group. (C) The quantification of TNF‐α in hippocampus of mice in each group. (D) The quantification of MMP8 in hippocampus of mice in each group. (E) The quantification of IL‐6 in cortex of mice in each group. (F) The quantification of TNF‐α in cortex of mice in each group. (G) The quantification of MMP8 in cortex of mice in each group.

To elucidate the mechanisms through which gastrodin influences ECM remodeling and BBB function, western blot analysis was conducted to evaluate the expression levels of IL‐6, tumor necrosis factor‐alpha (TNF‐α), and matrix metallopeptidase 8 (MMP8). The findings indicated that both low and high doses of gastrodin administered via gavage significantly increased the expression of IL‐6, TNF‐α, and MMP8 in the hippocampal and cortical regions of AD mice, in comparison to the control AD group (Figure 7A–G). Conversely, no significant differences were observed in the expression levels of these markers in high‐dose gastrodin‐treated wild‐type mice relative to untreated wild‐type controls.

## 4 Discussion

The physical microenvironment of the brain is intricately associated with neurodegenerative diseases, yet it is often undervalued as a pivotal factor influencing the degeneration of the nervous system [45, 46, 47, 48]. Empirical evidence indicates that the viscoelastic properties of brain tissue decline with advancing age, brain injury, and the progression of neurological disorders [5, 49]. Additionally, magnetic resonance elastography has detected localized reductions in tissue stiffness within the frontal, temporal, parietal, and sensorimotor regions in individuals diagnosed with AD [4, 45]. Prior studies have shown that the viscoelastic properties of the hippocampus deteriorate as AD progresses, with individual differences in hippocampal viscoelasticity being significantly associated with memory function [50]. Furthermore, the reduction in brain tissue viscoelasticity contributes to cerebral softening, which is intricately associated with the onset of demyelination [51, 52]. The softening of brain tissue may lead to abnormal growth and retraction of retinal ganglion cell axons, resulting in deviations from their normal pathways and consequently impairing their directionality and plasticity [53, 54, 55]. However, these changes in the physical microenvironment are not uniformly distributed throughout the brain; instead, they exhibit variability across different brain regions, specific pathological sites, and even between sexes [3]. In our preliminary investigations, we observed a reduction in the viscoelastic properties of brain tissue in AD model mice (3xTg) within both the hippocampal and cortical regions, as compared to age‐matched wild‐type mice, thereby corroborating findings previously reported in human brains (Figure S1). This study demonstrated that a four‐week regimen of intragastric gastrodin administration in 3xTg model mice led to an enhancement in tissue viscoelasticity in the drug intervention group. This increase effectively counteracted the reduction observed in the non‐drug intervention group, aligning the viscoelasticity levels more closely with those of age‐matched wild‐type mice. These results indicate that gastrodin influences the viscoelastic properties of brain tissue via specific molecular mechanisms.

The quintessential pathological characteristic of AD is the hyperphosphorylation of tau protein [56]. This process is modulated by a multitude of factors, notably the mechanical viscoelastic properties of the physical microenvironment and inflammatory mediators, which can influence its epigenetic regulation. Molecular dynamics simulations have demonstrated that modifications in the mechanical stress environment of tau influence its phosphorylation and dephosphorylation processes, as well as induce alterations in its strain rate‐dependent structural characteristics [57]. This indicates that changes in the microscopic physical environment can significantly impact the biological properties of tau. The tau protein plays a critical role in preserving axonal morphology and function through its interaction with and stabilization of microtubules [58]. However, when tau undergoes hyperphosphorylation, its binding affinity for microtubules diminishes, resulting in reduced axonal stability and increased susceptibility to abnormal breaks and deformations [59]. Furthermore, hyperphosphorylation of tau interferes with the neuronal transport system, impeding the intracellular movement of neurotransmitters, organelles, and nutrients, thereby adversely affecting normal neuronal function [60].

In this study, we observed that administration of Gastrodin resulted in the reversal of the viscoelastic properties of the brain tissue matrix, which was associated with a reduction in the phosphorylation levels of tau proteins (specifically tau S396, tau S356, and tau Thr231) in the treated mice. This biochemical alteration was accompanied by an increase in neuronal complexity, an enhancement in dendritic spine density, and an augmented release of synaptic vesicles. The initial findings demonstrated a significant reduction in the protein levels of phosphorylated tau at sites p‐S396, p‐S356, and Thr231 in the hippocampus and cortex at the low‐dose gastrodin treatment compared to the AD control group. However, the high‐dose group showed a lack of significant differences at the p‐S356 and Thr231 sites, which prompted us to further investigate potential mechanisms contributing to this discrepancy. Our data suggest that the response to gastrodin is not linear and may exhibit dose‐dependent characteristics. Specifically, at lower doses, gastrodin appears to effectively reduce tau phosphorylation at the targeted sites, possibly due to its ability to enhance the neuroprotective signaling pathways, such as the inhibition of glycogen synthase kinase‐3 beta (GSK‐3β) and extracellular signal‐regulated kinases (ERK), both of which have been implicated in tau hyperphosphorylation in AD models [61, 62]. These pathways are known to regulate tau phosphorylation at multiple sites, including p‐S396 and Thr231, and their modulation may contribute to the observed reduction in tau phosphorylation at these sites. This dose–response relationship suggests that optimizing the dosage of gastrodin could be crucial for maximizing its therapeutic potential in AD treatment. Future studies will focus on refining the dosing regimen, taking into account pharmacokinetic factors, and assessing the long‐term effects of gastrodin treatment on tau pathology and cognitive function in transgenic AD models [63, 64]. Furthermore, there was an upregulation in the expression of synaptic proteins, including SNAP25, PSD95, and SYP. The significant impact of the physical microenvironment's properties, including stiffness and viscoelasticity, on synaptic plasticity and adaptability may account for these observations. For example, alterations in environmental viscoelasticity can modulate morphological changes, synaptogenesis and pruning, postsynaptic signaling, and synaptic plasticity.

Furthermore, alterations in mechanical forces due to variations in the physical microenvironment significantly influence synaptic morphology and function [46, 65, 66]. These mechanical forces are capable of modulating synapse formation and elimination, the mechanical stability of synapses, synaptic plasticity, and postsynaptic signaling. Such modulation may also contribute to the mitigation of tau pathology [67]. The attenuation of tau hyperphosphorylation enhances the intracellular transport mechanisms within neurons, thereby promoting a partial restoration of synaptic functionality and subsequently improving cognitive performance and spatial recognition capabilities in mice treated with Gastrodin. Furthermore, the ECM is integral to preserving the viscoelastic properties of brain tissue, which are essential for critical processes including neuronal migration, axonal growth, and synaptogenesis. Recent research provides additional evidence suggesting that alterations in the composition and structure of the ECM are closely associated with the initiation and progression of neurological diseases [45, 68]. All tissue cells, including those in the brain, are subject to endogenous physical forces such as hydrostatic pressure, shear stress, compressive forces, and tension. Modifications in the ECM are crucial for alterations in endogenous physical mechanics, as cells sustain homeostasis through “mechanical interactions” with the ECM by adapting their behavior and remodeling their microenvironment [69, 70, 71]. Disruption of these “mechanical interactions” can precipitate the onset and progression of diseases. The physical properties of the ECM, including its shape, dimensions, viscoelasticity, microstructure, and pore size, exert a significant influence on the functioning of the nervous system, although the precise mechanisms underlying these effects warrant further investigation [72, 73]. The ECM detects alterations in tissue mechanical viscoelasticity via mechanosensor factors, with integrins and adhesion proteins serving as mechanosensing elements that link to the cytoskeleton, such as actin, thereby facilitating the transmission of mechanical signals between the intracellular environment and the extracellular ECM [74, 75].

Comprehensive research has identified FERMT2 as a risk gene for AD, a finding substantiated by multiple GWAS and PRS [48]. SNPs within this gene have been associated with both fAD and LOAD, with the most pronounced association observed during the mild cognitive impairment stage. Building upon this discovery, subsequent research has demonstrated that FERMT2 is predominantly expressed in astrocytes, a finding that our study supports (Figure S2). Moreover, FERMT2 has been confirmed to function as a mechanosensor in human lung cancer tissues, where it interacts with the proline synthesis enzyme PYCR1 in response to alterations in ECM stiffness. This interaction leads to elevated PYCR1 levels and augmented proline synthesis, which in turn promote collagen synthesis and remodeling [32, 76]. Consequently, we propose the hypothesis that the integrin adhesion receptor FERMT2 might similarly participate in an interactive dialogue concerning alterations in mechanical viscoelasticity and ECM remodeling within the brain, thereby influencing ECM‐related biological processes and the progression of AD. Our research, utilizing artificial intelligence to simulate drug–protein interactions alongside CETSA experiments, demonstrated that Gastrodin exhibits a high binding affinity for FERMT2. Immunofluorescence staining indicated that following Gastrodin treatment in 3xTg mice, the fluorescence intensity of FERMT2, specifically expressed in astrocytes, was reduced. In a separate study currently under submission, we observed that targeted downregulation of astrocytic FERMT2 via adeno‐associated virus reversed brain matrix viscoelasticity, consistent with the trends observed following Gastrodin administration. Therefore, we hypothesize that the astrocyte‐specific expression of FERMT2 could potentially serve as a therapeutic target for Gastrodin, facilitating its function in modulating brain matrix viscoelasticity. Subsequent experimental analyses demonstrated that in 3xTg mice treated with the drug, the expression levels of ECM components, specifically collagen type I and collagen type IV, were significantly elevated in both hippocampal and cortical brain tissues compared to the control group. Collagen plays a crucial role in the viscoelastic properties of the brain matrix, with type I collagen being the predominant form within the ECM. It is primarily located in the basement membrane and the surrounding ECM, where it contributes to the maintenance of ECM structure and mechanical integrity. The observed upregulation of type I collagen expression substantiates the hypothesis that Gastrodin may have the potential to restore the viscoelastic properties of the brain matrix [77, 78].

Collagen type IV is primarily localized within the basement membranes of cerebral vasculature and among neural supportive cells, with its expression levels being intricately associated with the integrity of the BBB and the structural stability of neural tissue [79]. Empirical evidence indicates that collagen type IV facilitates the adhesion and proliferation of endothelial cells through its interaction with integrin receptors on the cell surface [80]. This interaction not only preserves the morphology of endothelial cells but also enhances intercellular tight junctions, thereby reducing permeability and ensuring the selective permeability characteristic of the BBB. Additionally, studies have demonstrated that collagen type IV plays a regulatory role in endothelial cell function by activating the PI3K/Akt and MAPK signaling pathways, which in turn influence the homeostasis of the BBB and its response to external stimuli, including inflammation [81, 82]. Consequently, we hypothesize that Gastrodin may facilitate the remodeling of ECM components, particularly collagen, through specific molecular targets, thereby impacting the viscoelastic properties of the brain matrix.

This study demonstrated that Gastrodin treatment effectively restored the integrity and permeability of the BBB in a murine model of AD by reversing alterations in brain matrix viscoelasticity. Existing research underscores the significance of the ECM physical properties, including stiffness and viscoelasticity, in preserving BBB functionality. An increase in matrix stiffness is frequently linked to enhanced endothelial cell proliferation and migration, potentially resulting in BBB compromise. The viscoelastic properties of the ECM play a critical role in the development and preservation of intercellular connections among endothelial cells, including tight junctions and adherens junctions. Tight junctions are crucial for maintaining the selective permeability characteristic of the BBB [83, 84]. Alterations in the matrix's viscoelasticity can lead to changes in the morphology and connectivity of endothelial cells, thereby influencing the integrity of intercellular barrier functions [10, 85]. Furthermore, the viscoelasticity of the matrix may affect endothelial cell functionality by modulating cellular signaling pathways [86, 87]. For instance, the physical characteristics of the ECM have the potential to activate integrins and other cell membrane receptors, thereby initiating intracellular signaling pathways, such as the RhoA/ROCK pathway [88, 89]. This activation subsequently influences cellular morphology and the stability of intercellular junctions. The correlation between alterations in matrix viscoelasticity and the integrity and permeability of the BBB is notably significant in the context of neurodegenerative diseases [90, 91].

Concurrently, this study also noted alterations in the expression levels of matrix MMP8, TNF‐α, and IL‐6 subsequent to Gastrodin intervention. MMP8 is a matrix metalloproteinase predominantly involved in the degradation of collagen and other ECM components [92]. Evidence suggests that increased levels of MMP8 are linked to neuroinflammation and modifications in the ECM, with excessive expression potentially resulting in ECM changes that affect neuronal survival and function [93]. In AD, elevated levels of TNF‐α and IL‐6 are frequently observed and are intricately associated with neuroinflammation [94]. The secretion of TNF‐α and IL‐6 within the neuroinflammatory milieu not only fosters the recruitment of inflammatory cells but also exacerbates the progression of AD by affecting ECM remodeling and compromising the integrity of the BBB, thereby perpetuating a deleterious cycle [95, 96, 97]. Furthermore, elevated concentrations of TNF‐α and IL‐6 may contribute to the accumulation of beta‐amyloid and the aberrant phosphorylation of tau protein, thus expediting neuronal damage and cell death [98, 99].

Consequently, the interplay among inflammatory factors, ECM remodeling, and the BBB constitutes a dynamic and intricate network. Previous research has established that the viscoelastic properties of the ECM undergo significant alterations in AD; these modifications can facilitate the release of inflammatory mediators, which in turn exacerbate BBB disruption. Furthermore, inflammatory factors can modify the composition of the ECM, as demonstrated in this study with collagen types I and IV, thereby impacting its viscoelasticity and BBB functionality. This underscores the interconnected nature of these three components. Our study demonstrates that Gastrodin has the potential to reverse the deterioration of brain matrix viscoelasticity, potentially attributable to its function as a natural inhibitor of the mechanosensor FERMT2, alongside its suppressive effects on MMP8, TNF‐α, and IL‐6. This synergistic mechanism in AD treatment may facilitate the remodeling of ECM components, specifically collagen types I and IV, thereby restoring matrix viscoelasticity, reducing BBB permeability, and enhancing the expression of vascular tight junction proteins. As a result, there is a decrease in tau protein hyperphosphorylation in AD mice, which aids in the restoration of synaptic integrity and improves cognitive function.

## 5 Conclusion

Gastrodin mitigates tau hyperphosphorylation in 3xTg‐AD mice, thereby ameliorating synaptic damage and enhancing cognitive function. To investigate the underlying mechanisms, we discovered that gastrodin can reverse the reduction in brain matrix viscoelasticity in 3xTg‐AD mice and induce the remodeling of ECM components. This effect may be attributed to the AD risk gene and mechanotransduction factor FERMT2, which is predominantly expressed in astrocytes and represents a potential target of gastrodin. Gastrodin exhibits a high binding affinity for FERMT2 and can specifically inhibit its expression in astrocytes, leading to ECM remodeling and reversing the decline in matrix viscoelasticity, alongside the functional restoration of the BBB and alterations in the expression of neuroinflammatory factors such as TNF‐α, IL‐6, and metalloproteinase 8, it is further demonstrated that matrix viscoelasticity is intricately linked to the interaction between BBB permeability and neuroinflammation.

## Author Contributions

X.Q. and L.W. designed the experiments. L.W. and B.L. performed experiments; Y.W., Y.P., and A.Z. carried out the data collection and collation. Y.W. and T.S. analyzed the data. L.W. and X.Q. conducted the experiment and wrote the manuscript draft. All the authors edited the manuscript and approved the manuscript before submission.

## Ethics Statement

Animal experiments were approved by Guizhou Medical University's Animal Care and Use Committee (approval No. 2304543) and followed ARRIVE 2.0 guidelines.

## Conflicts of Interest

The authors declare no conflicts of interest.

## Supporting information


Data S1.

**Table S1.** Antibodies used in this study.

**Table S2.** Chemicals and kits used in this study.

## Data Availability

The data that support the findings of this study are available on request from the corresponding author. The data are not publicly available due to privacy or ethical restrictions.
