# Supplementary material for: Gastrodin Alleviates Tau Pathology by Targeting the Alzheimer's Risk Gene FERMT2, Reversing the Reduction in Brain Viscoelasticity
Source: CNS Neurosci Ther. 2025 Mar 22;31(3):e70283. doi: 10.1111/cns.70283 (PMC11928745; doi:10.1111/cns.70283)
Supplement: Supplementary file 1 — Data S1. [file CNS-31-e70283-s002.docx]

**Supplementary Figure.**

**
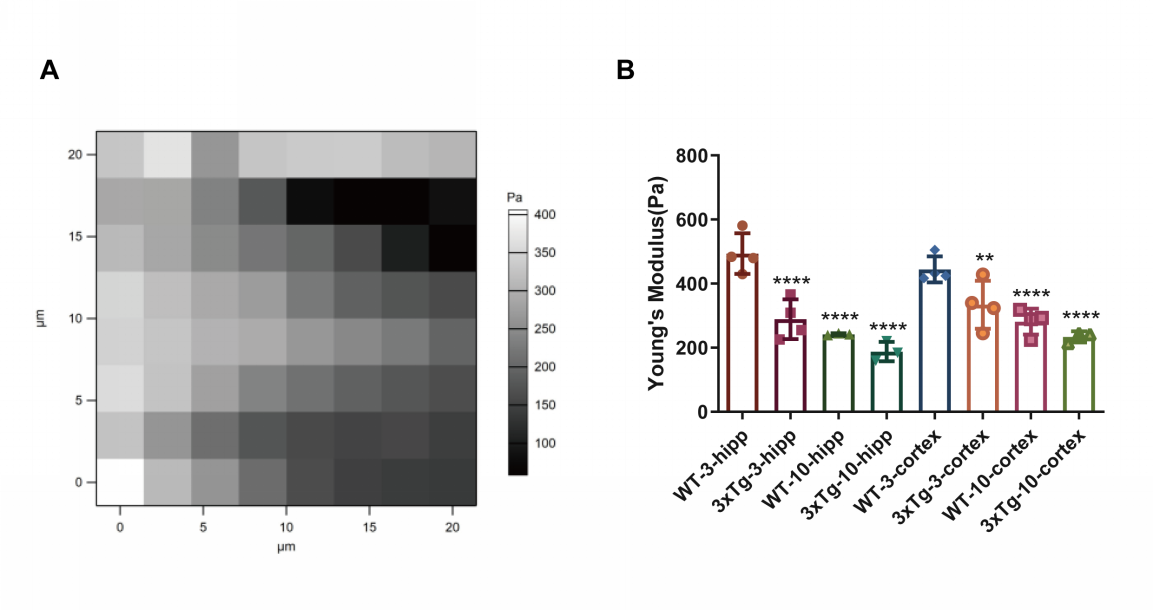
**

As illustrated in the figure, Young's modulus in the brains of 3xTg-AD model rats exhibits a decline with advancing age, indicating a reduction in matrix viscoelasticity. ^**^P<0.001, ^****^P<0.0001.

**Fig.S1**


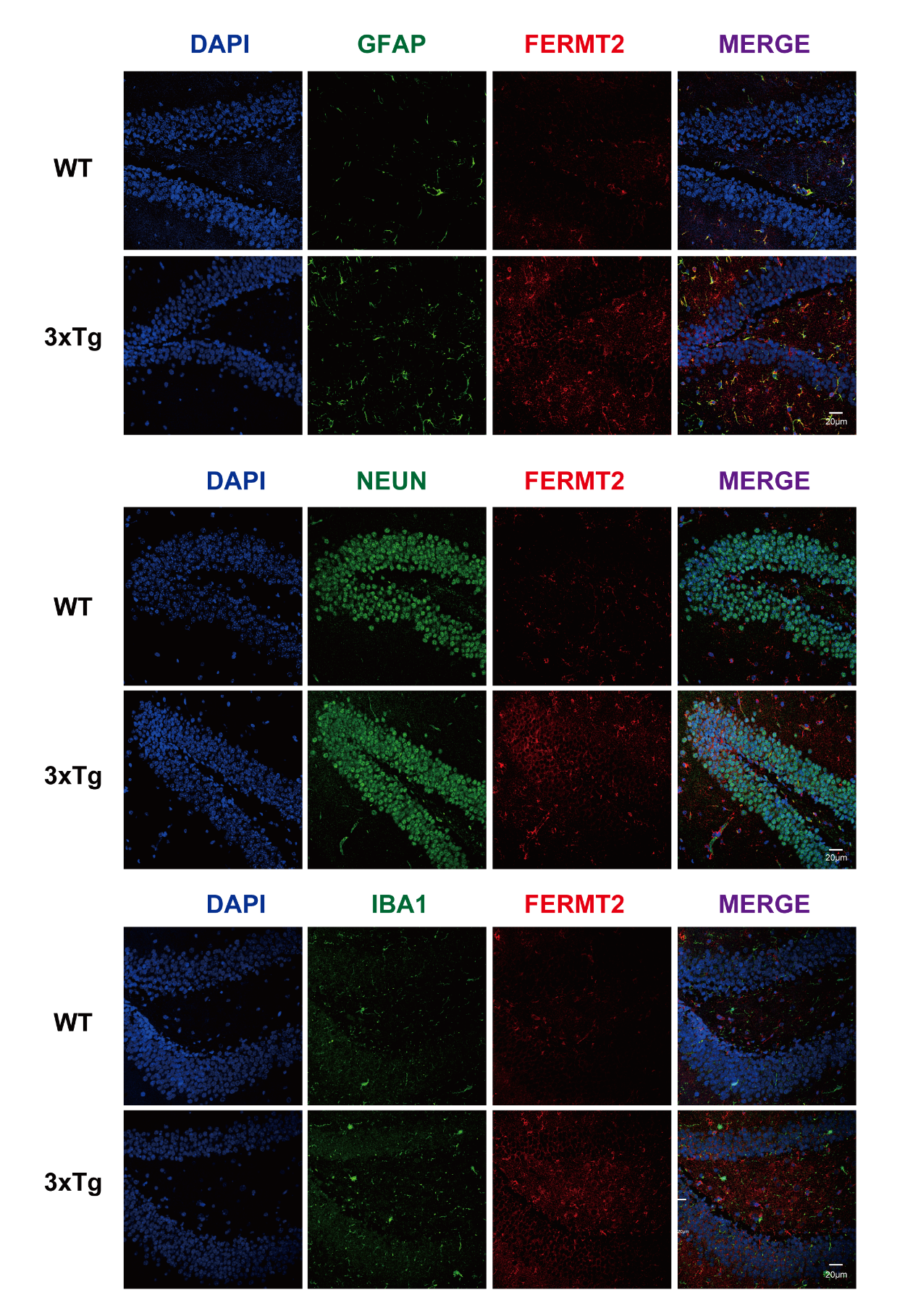


**Fig.S2**

As illustrated in the figure, immunofluorescence staining revealed that FERMT2 predominantly colocalized with astrocytes, thereby confirming its primary expression within these cells.
