# Supplementary material for: Gastrodin Alleviates Tau Pathology by Targeting the Alzheimer's Risk Gene FERMT2, Reversing the Reduction in Brain Viscoelasticity
Source: CNS Neurosci Ther. 2025 Mar 22;31(3):e70283. doi: 10.1111/cns.70283 (PMC11928745; doi:10.1111/cns.70283)
Supplement: Supplementary file 2 — Table S1. Antibodies used in this study. [file CNS-31-e70283-s003.docx]

**Supplemental Table 1. Antibodies used in this study.**

| **Antibody** | **Host** | **Specificity** | **WB/IF**  **Dilution** | **Sources** | **Catalog No** |
| --- | --- | --- | --- | --- | --- |
| Anti-beta Amyloid 1-42 | r | - | 1:1000 | Abcam | #ab180956 |
| Anti-FERMT2 | r | - | 1:2000 | Abcam | #ab194967 |
| Anti-FERMT2 | r | - | 1:1000 | Proteintech | 11453-1-AP |
| Anti-MMP8 | r | - | 1:1000 | Proteintech | 17874-1-AP |
| Anti-MMP8 | m | - | 1:50 | SANTA | sc-514803 |
| Anti-Tau S396 | r | p-Tau | 1:1000 | Abcam | #ab32057 |
| Anti-Tau S356 | r | p-Tau | 1:1000 | Abcam | #ab75603 |
| Anti-Tau Thr231 | r | p-Tau | 1:5000 | Abcam | #ab151559 |
| Anti-Tau5 | m | - | 1:1000 | Abcam | #ab80579 |
| Anti-SNAP25 | r | - | 1:5000 | Abcam | #ab5666 |
| Anti-synaptophysin | r | - | 1:10000 | Abcam | #ab32127 |
| Anti-PSD95 | r | - | 1:2000 | Abcam | #ab18258 |
| Anti-Collagen1 | r | - | 1:1000 | Abcam | #ab138492 |
| Anti-Collagen4 | r | - | 1:1000 | Affinity | #AF0510 |
| Anti-IL-6 | r | - | 1:1000 | Affinity | #DF6087 |
| Anti-TNF-α | r | - | 1:500 | Affinity | #AF7014 |
| Anti-β-tubulin | m | - | 1:5000 | Beyotime | #21463 |
| Anti-β-actin | m | - | 1:25000 | Affinity | #T0022 |
| Anti- Fibrinogen  alpha chain | r | - | 1:50 | Abcam | #ab92572 |
| Anti-CD31 | m | - | 1:500 | Proteintech | 66065-2-lg |
| Anti-rabbit IgG (H+L) (HRP) | g | - | 1:5000 | Thermo Fisher | #32260 |
| Anti-mouse IgG (H+L) (HRP) | g | - | 1:5000 | Thermo Fisher | #32230 |
| Anti-Mouse IgG (H+L) Cyanine3 | g | - | 1:200 | Thermo Fisher | #31188 |
| Anti-Rabbit IgG (H+L) Alexa Fluor™546 | d | - | 1:200 | Thermo Fisher | #31466 |

T: total; P: phosphorylated; r: rabbit; m: mouse; g: goat; d: donkey; IF: immunofluorescence; WB: western blot;
