# Supplementary material for: Gastrodin Alleviates Tau Pathology by Targeting the Alzheimer's Risk Gene FERMT2, Reversing the Reduction in Brain Viscoelasticity
Source: CNS Neurosci Ther. 2025 Mar 22;31(3):e70283. doi: 10.1111/cns.70283 (PMC11928745; doi:10.1111/cns.70283)
Supplement: Supplementary file 3 — Table S2. Chemicals and kits used in this study. [file CNS-31-e70283-s001.docx]

**Supplemental Table 2. Chemicals and kits used in this study.**

| **Chemical/kit** | **Sources** | **Catalog No** |
| --- | --- | --- |
| Methylpropene gelatin | Engineering For Life | EFL-GM-60 |
| Portable curing light source | Engineering For Life | EFL-LS-1601 |
| β-Amyloid (1-42) | GL Biochem (shanghai) Co., Ltd | #GLS-52487 |
| C57BL/6 J mice and 3xTg Mice | Jiangsu Anipe Biolaboratory Inc (Jiangsu, China) |  |
| DAPI solution | Solarbio | # C0065 |
| RIPA buffer(high) | Solarbio | #R0010 |
| Protease inhibitor mixture | Solarbio | #P6730 |
| Fetal Bovine Serum | Thermo Fisher | #10099141C |
| HFIP | Sigma-Aldrich | #105228 |
| DMSO | Sigma-Aldrich | #D2650 |
| PEG 300 | Sigma-Aldrich | #91462 |
| DAB color development kit | ZSBG-BIO | ZLI-9017 |
| Triton™ X-100 | Sigma-Aldrich | #X100 |
| Pierce™ BCA Protein Test Kit | Thermo Fisher | #23227 |
| FD Rapid Golgi staining ™kit | FD | #PK401A |
